# Landmark Detection in Cardiac MRI by Using a Convolutional Neural Network

**DOI:** 10.1148/ryai.2021200197

**Published:** 2021-07-14

**Authors:** Hui Xue, Jessica Artico, Marianna Fontana, James C. Moon, Rhodri H. Davies, Peter Kellman

**Affiliations:** From the National Heart, Lung, and Blood Institute, National Institutes of Health, 10 Center Dr, Bethesda, MD 20892 (H.X., P.K.); Barts Heart Centre, National Health Service, London, England (J.A., J.C.M., R.H.D.); and National Amyloidosis Centre, Royal Free Hospital, London, England (M.F.).

**Keywords:** Cardiac, Heart, Convolutional Neural Network (CNN), Deep Learning Algorithms, Machine Learning Algorithms, Feature Detection, Quantification, Supervised Learning, MR Imaging

## Abstract

**Purpose:**

To develop a convolutional neural network (CNN) solution for landmark detection in cardiac MRI (CMR).

**Materials and Methods:**

This retrospective study included cine, late gadolinium enhancement (LGE), and T1 mapping examinations from two hospitals. The training set included 2329 patients (34 089 images; mean age, 54.1 years; 1471 men; December 2017 to March 2020). A hold-out test set included 531 patients (7723 images; mean age, 51.5 years; 323 men; May 2020 to July 2020). CNN models were developed to detect two mitral valve plane and apical points on long-axis images. On short-axis images, anterior and posterior right ventricular (RV) insertion points and left ventricular (LV) center points were detected. Model outputs were compared with manual labels assigned by two readers. The trained model was deployed to MRI scanners.

**Results:**

For the long-axis images, successful detection of cardiac landmarks ranged from 99.7% to 100% for cine images and from 99.2% to 99.5% for LGE images. For the short-axis images, detection rates were 96.6% for cine, 97.6% for LGE, and 98.7% for T1 mapping. The Euclidean distances between model-assigned and manually assigned labels ranged from 2 to 3.5 mm for different landmarks, indicating close agreement between model-derived landmarks and manually assigned labels. For all views and imaging sequences, no differences between the models’ assessment of images and the readers’ assessment of images were found for the anterior RV insertion angle or LV length. Model inference for a typical cardiac cine series took 610 msec with the graphics processing unit and 5.6 seconds with central processing unit.

**Conclusion:**

A CNN was developed for landmark detection on both long- and short-axis CMR images acquired with cine, LGE, and T1 mapping sequences, and the accuracy of the CNN was comparable with the interreader variation.

**Keywords:** Cardiac, Heart, Convolutional Neural Network (CNN), Deep Learning Algorithms, Machine Learning Algorithms, Feature Detection, Quantification, Supervised Learning, MR Imaging

*Supplemental material is available for this article.*

Published under a CC BY 4.0 license.

## Graphical abstract

**Figure:**
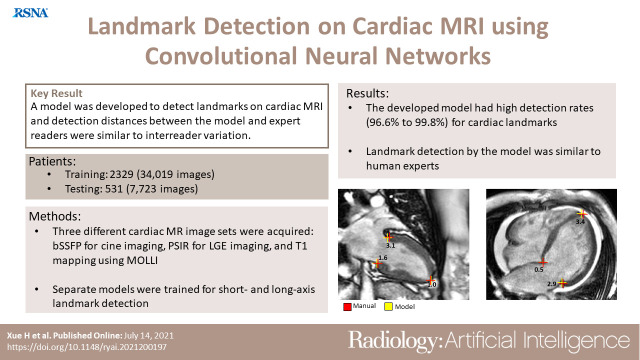

**Summary**

A convolutional neural network (CNN) was developed for labeling landmarks on long- and short-axis cardiac MR images acquired by using cine, late gadolinium enhancement, and T1 mapping sequences, and the performance of CNN labeling was comparable with that of manual labeling.

**Key Points**

■ The developed model achieved a high detection rate for cardiac landmarks (ranging from 96.6% to 99.8%) on the test dataset.
■ Comparison of right ventricular insertion angle and left ventricular length measurements between the developed model and experts was similar for different cardiac MRI views.
■ Models were integrated with MRI scanners by using Gadgetron InlineAI, with less than 1 second of model inference time.

## Introduction

Cardiac MRI (CMR) is emerging as a mainstream modality for imaging the cardiovascular system for diagnosis and intervention. CMR has advanced beyond the scope of imaging the anatomy and can be used to acquire comprehensive quantitative measures of the myocardium. These include relaxometry T1, T2, and T2* measures (1,2) for the assessment of fibrosis, edema, and iron as well as for assessment of tissue composition for the fat fraction (3) and include physiologic measures for mapping of myocardial perfusion (4,5) and blood volume (6). These capabilities open new opportunities and simultaneously place new demands on image analysis and reporting. A fully automated solution brings increased objectivity and reproducibility and higher patient throughputs.

Research in the field of automated analysis and reporting of CMR is continuing to advance. In clinical practice, manual delineation by cardiologists remains the main approach for quantifying cardiac function, viability, and tissue properties (7). A recent study showed that a detailed manual analysis by an expert can take anywhere from 9 to 19 minutes (8). Thus, automated image delineation could help reduce the time needed for image assessment.

Deep learning models, convolutional neural networks (CNNs) in particular, have been developed to automate CMR analysis. Cardiac cine images can be automatically analyzed by using CNNs to measure the ejection fraction and other parameters with a performance level matching that of expert readers (9), and CNN measurements have demonstrated improved reproducibility in multicenter trials (8,10). Cardiac perfusion images have been successfully analyzed and reported on the MRI scanners (11) by using CNNs. CNNs have also been developed to quantify left ventricular (LV) function in multivendor, multicenter experiments (12). Additionally, deep learning CNNs have been developed for automatic myocardial scar quantification (13). Current research has focused on automating the time-consuming processes of segmenting the myocardium.

To achieve automated analysis and reporting of CMR, key landmark points must be located on the cardiac images. For example, right ventricular (RV) insertion points are needed to report quantitative maps with use of the standard American Heart Association sector model (7). For long-axis views, the ventricular length can be measured if the valve and apical points can be delineated. Variation in the LV length is a useful marker and has been shown to be the principal component of LV pumping in patients with chronic myocardial infarction (14). Furthermore, cardiac landmark detection can be useful on its own for applications such as automated imaging section planning.

In this study, we developed a CNN-based solution for automatic cardiac landmark detection on CMR images. Detection was defined as the process of locating the key landmark points from CMR images acquired in both short- and long-axis views. The proposed CNN model predicts the spatial probability of a landmark on the image. The performance of the trained model was quantitatively evaluated by comparing the success rates between CNN labeling and manual labeling and by computing the Euclidean distance between manually derived and model-derived landmarks. To evaluate the feasibility of using models for CMR reporting, the model-derived and manually derived angle of the anterior RV insertion point (A-RVI) and LV length were used. To demonstrate their clinical feasibility, the trained CNN models were integrated with MRI scanners by using Gadgetron InlineAI (15) and were used to automatically measure the LV length from long-axis cine images. The developed model has the potential to reduce the amount of time needed for CMR image assessment.

## Materials and Methods

### Study Design

**Figure 1:**
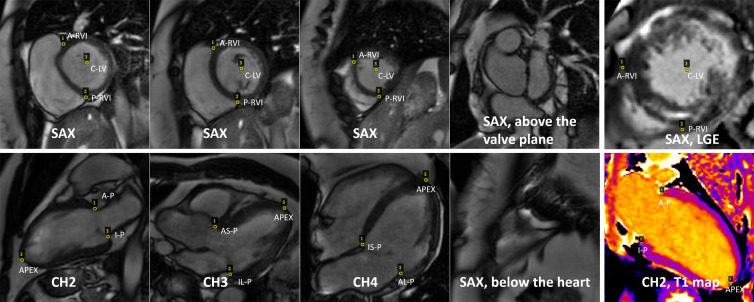
Example of cardiac MR images with landmarks. Three short-axis (SAX) views are shown on the top row. The first three images of the second row show examples of long-axis two-chamber (CH2), three-chamber (CH3), and four-chamber (CH4) views. The anterior point (A-P) and inferior point (I-P) were depicted on the two-chamber view. The inferolateral point (IL-P) and anteroseptal point (AS-P) were depicted on the three-chamber view, and the inferoseptal point (IS-P) and anterolateral point (AL-P) were depicted on the four-chamber view. The apical point (APEX) was depicted on all long-axis views. For the short-axis images, the anterior right ventricular (RV) insertion point (A-RVI), posterior RV insertion point (P-RVI), and left ventricular (LV) center point (C-LV) were depicted. Note that for some SAX sections (the rightmost column), no landmarks can be identified. The last column gives examples of late gadolinium enhancement (LGE) images and T1 maps. Transfer learning was applied to detect landmarks by using these imaging applications.

The developed CNN was designed to detect landmarks on both long-axis series (two chamber, three chamber, and four chamber) and short-axis series (Fig 1). The following points were detected on different views: *(a)* short-axis view, the A-RVI, posterior RV insertion point (P-RVI), and LV center point (C-LV); *(b)* two-chamber view, the anterior and inferior points; *(c)* three-chamber view, the inferolateral and anteroseptal points; *(d)* four-chamber view, the inferoseptal and anterolateral points; and *(e)* long-axis view, the apical point. The trained CNN models were tested on cardiac cine images, late gadolinium enhancement (LGE) images, and T1 maps acquired by using a modified Look-Locker inversion recovery (MOLLI) imaging sequence (1,16).

### Data Collection

In this retrospective study, a dataset was assembled from two hospitals. All cine and LGE examinations were performed at the Barts Heart Centre (London, England), and all T1 MOLLI images were acquired at the Royal Free Hospital (London, England). Both long- and short-axis views were acquired for cine and LGE series. For T1 mapping, one to three short-axis sections were acquired per patient. The data used in this study were not used in prior publications.

Data were acquired with the required ethical and/or secondary audit use approvals or guidelines (as per each center), which permitted retrospective analysis of anonymized data without requiring written informed consent for secondary usage for the purpose of technical development, protocol optimization, and quality control. Institutions acquiring data were in the United Kingdom and were not subject to the Health Insurance Portability and Accountability Act. All data were anonymized and delinked for analysis, with approval being provided by the local Office of Human Subjects Research (exemption 13156). Appendix E1 (supplement) provides information about patient inclusion criteria.

Table 1 summarizes the training and test datasets. For training, a total of 34 089 images from 2329 patients (mean age, 54.1 years; 1471 men) were included—29 214 cine, 3798 LGE, and 1077 T1 images. Cine training data were acquired from three time periods in 2017, 2018, and 2020, as listed in Table 1. All patients who underwent LGE imaging also underwent cine imaging. Data acquisition in every imaging period was consecutive. The test set consisted of 7723 images from 531 consecutive patients (mean age, 51.5 years; 323 men). The test data were acquired between May and June 2020. There was no overlap between the training and test sets. No test data were used in any way during the training process and was a completely held-out dataset.

**Table 1:**
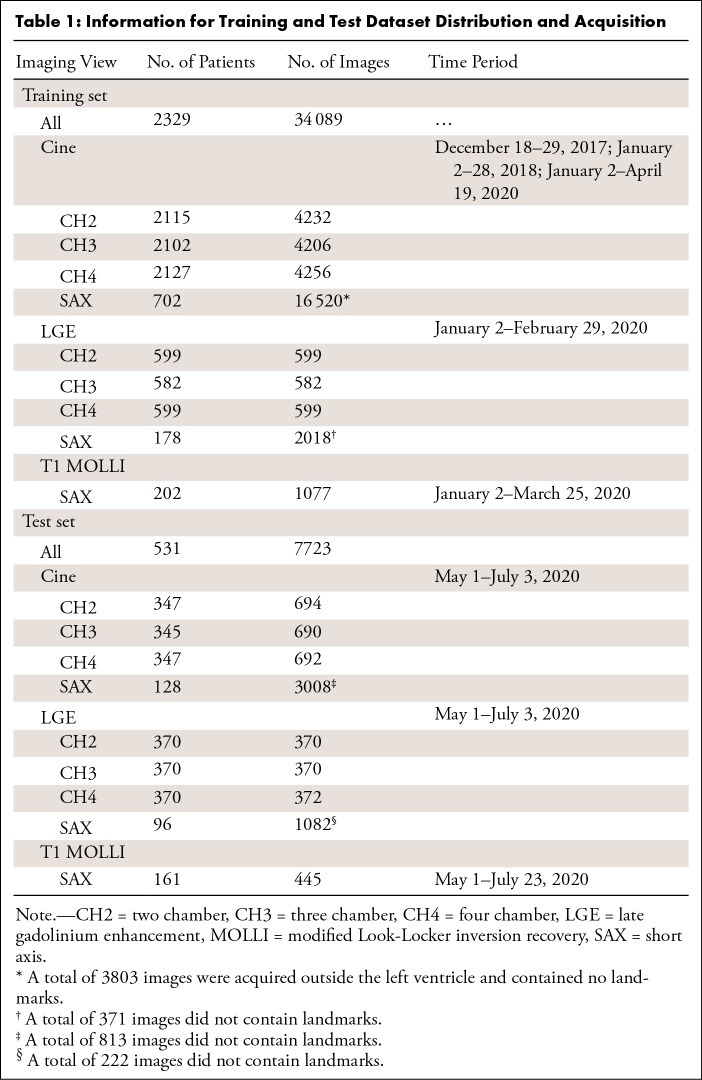
Information for Training and Test Dataset Distribution and Acquisition

### CMR Acquisition

Images were acquired by using both 1.5-T (four Magnetom Aera scanners, Siemens Healthineers) and 3-T (one Magnetom Prisma, Siemens Healthineers) MRI scanners. In the training set, 1790 patients were imaged with 1.5-T scanners and 539 patients were imaged with 3-T scanners. In the test set, 462 patients were imaged with 1.5-T MRI and 69 were imaged with 3-T MRI. Typically, 30 cardiac phases were reconstructed for each heartbeat for every cine scan. For training and testing purposes, the first phase (typically the end-diastolic phase) and the end-systolic phase were selected. Given that there was a large number of patients, these acquired cardiac phases represent a sufficiently broad variation. For those who underwent contrast-enhanced studies, the gadolinium-based contrast agent (gadoterate meglumine [Dotarem, Guerbet]) was administered at 4 mL/sec at a dose of 0.05 mmol/kg.

### Imaging Sequences

The imaging parameters for each sequence are shown in Table E1 (supplement).

***Balanced steady-state free precession cine imaging.—***Cine imaging was performed with retrospective electrocardiographic gating (30 cardiac phases were reconstructed) and twofold parallel imaging acceleration by using generalized autocalibrating partially parallel acquisition, or GRAPPA (17). For the short-axis acquisition, eight to 14 sections were typically used in order to cover the LV area.

***Phase-sensitive inversion recovery for LGE imaging.—***Phase-sensitive inversion recovery LGE imaging was performed by using a free-breathing sequence (18) to enable coverage of the entire LV area while applying respiratory motion correction and averaging. Phase-sensitive LGE reconstruction (19) was used to achieve insensitivity to the inversion time. A previous study (20) showed that this free-breathing technique is more robust against respiratory motion and resulted in improved LGE image quality.

***T1 mapping with use of MOLLI.—***T1 mapping was performed with a previously published MOLLI protocol (1). The sampling strategy was 5s(3s)3s for precontrast T1 imaging and 4s(1)3s(1s)2s for postcontrast imaging. A retrospective motion correction algorithm (21) was applied to MOLLI images and then went through T1 fitting (22) to estimate per-pixel maps.

### Data Preparation and Labeling

Because the acquired field of view may have varied among patients, all images were first resampled to a fixed 1-mm^2^ pixel spacing and were padded or cropped to 400 × 400 pixels before input into the CNN. This corresponds to a processing field of view of 400 mm^2^, which is large enough to cover the heart, as the MRI technicians generally positioned each patient so that the heart would be close to the center of the field of view. The use of cine MRI often causes a shadow across the field of view (Fig E1 [supplement]), as the tissue that is further away from receiver coils at the chest and spine will have a reduced signal intensity due to the inhomogeneity of the surface coil receiver sensitivity. To compensate for this shading, for every cine image in the dataset, a surface coil inhomogeneity correction algorithm (23) was applied to estimate the rate of the slowly varying surface coil sensitivity, which was used to correct this inhomogeneity. During training, either the original cine image or the corrected image was fed into the network, and *P* = .5 was the probability of selecting the original version. This served as one data augmentation step. Additional details on other data augmentation procedures are found in Appendix E2 (supplement).

One reader (H.X., with 9 years of experience in CMR research and 3 years of experience in deep learning) manually labeled all images for training and testing. A second reader (J.A., with 3 years of experience in CMR clinical reporting) was invited to label part of the test dataset to assess interreader variation. J.A. labeled 1100 images (cine and LGE: 100 images for every long-axis view, 200 images for every short-axis view; 100 images for every T1 map). The Visual Geometry Group Image Annotator software (*https://www.robots.ox.ac.uk/~vgg/software/via/*), or VIA, was used by both readers for the manual labeling of landmarks. The data labeling took approximately 150 hours in total. Table 1 shows the training and test datasets.

### Model Development

**Figure 2:**
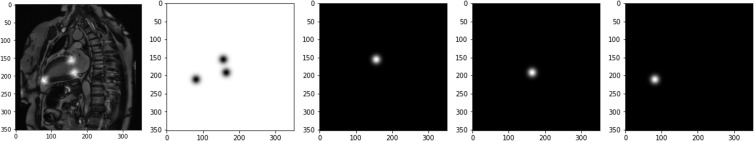
The landmark detection problem can be reformulated as a semantic segmentation problem. Every landmark point on the two-chamber image on the left can be convolved with a Gaussian kernel and converted into a spatial probability map or heatmap (upper row, from left to right: probability for background, anterior valve point, inferior valve point, and apical point). Unlike in the binary detection task in which the target is a one-hot binary mask, loss functions working on continuous probability such as the Kullback-Leibler divergence are needed.

The landmark detection problem was formulated as a heatmap (24). As shown in Figure 2, every landmark point was convolved with a Gaussian kernel (σ = 4.0 pixels), and the resulting blurred distribution represents the spatial probability of the landmark. Detecting three landmarks was equivalent to a semantic segmentation problem for four classes (one background class and one object class for each landmark). Class labels for different landmarks were represented as channels in probability maps; thus, if there are three landmarks to be detected, there will be four heatmaps (three maps for three landmarks and one map for the background). Additional information on the heatmaps is provided in Appendix E3 (supplement).

### Model Training

**Figure 3:**
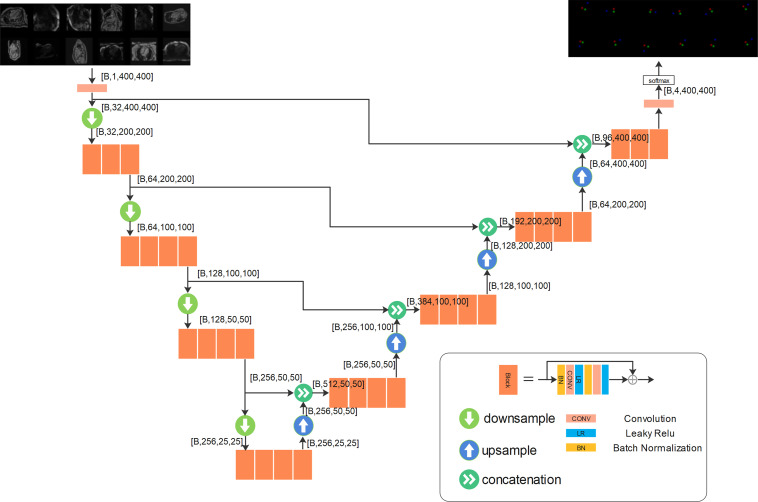
The backbone convolutional neural network developed for landmark detection has a U-Net structure. More layers can be inserted in both the downsampling branch and the upsampling branch, and more blocks can be inserted into each layer. The output layer outputs the per-pixel scores, which go through softmax function. For the landmark detection on long-axis images, data from three views were used together to train one model. As shown in the input, every minibatch was assembled by using randomly selected images from three views and was used for back propagation. A total of four layers with three or four blocks per layer were used in this experiment. The output tensor shapes were reported by using the format [B, C, H, W], where B is the size of the minibatch, and C is the number of channels, and H and W are the image height and width. Input images have one channel for image intensity, and the output has four channels for three landmarks and the background. The illustration for outputs plots three color-coded landmark channels and omits the background channel.

A variation of U-Net architecture was implemented (25,26) for heatmap detection. As shown in Figure 3, the network was organized as layers for different spatial resolutions. Specific details on the model architecture are described in Appendix E4 (supplement). The input to the model was a two-dimensional image (ie, to detect the landmarks from a time series of cine images, the model was applied to each two-dimensional image using the current model configuration).

In the data preparation step, all images were resampled and cropped to 400 × 400 pixels. The CNN output score tensor had dimensions of 400 × 400 × 4. To train the network, the Kullback-Leibler divergence was computed between the ground truth heatmap and the softmax tensor of the scores. Besides this entropy-based loss, the shape loss was further computed as the soft Dice ratio (27). The soft Dice ratio was computed as the product of two probability maps over their sum. The final loss was a sum of the entropy-based loss and the soft Dice ratio, which used both entropy-based information and region costs. This strategy of using a combined loss has previously been employed in deep learning segmentation and has been found to improve segmentation robustness (28,29).

For the long-axis views, all views were trained together as a multitask learning task. Because the number of images for each long-axis view was roughly equal, no extra data-rebalancing strategy was applied. Instead, every minibatch randomly selected from two-chamber, three-chamber, or four-chamber images, and refined network weights.

The data for training were split, with 90% of all patients for training and 10% for validation. The training and validation datasets were split on a per-study basis, such that there was no mixing of patients between the two datasets. The Adam optimizer was used, and the initial learning rate was 0.001, the β values were 0.9 and 0.999, and the ε value was 1 × 10^−8^. The learning rate was reduced by 2 whenever the cost function plateaued. Training lasted 50 epochs (approximately 4 hours), and the best model was selected as the one demonstrating the highest performance on the validation set. The CNN models were implemented by using PyTorch (30), and training was performed on a personal computer running Ubuntu 20.04 with four NVIDIA GeForce GTX 2080Ti graphics processing unit cards, each with 11 GB of random access memory. Data parallelization was used across multiple graphics processing unit cards to speed up training.

Because there were more cine images than LGE and T1 MOLLI images, a fine-tuning strategy was implemented by using transfer learning. For both long- and short-axis images, a model was first trained with the cine dataset and then fine-tuned with either the LGE or T1 training set. Transfer learning was implemented to first train the neural networks with the cine data as the pretrained model. The LGE or T1 data were used to fine-tune the pretrained model with a reduced learning rate (31). To perform the fine-tuning, the initial learning rate was set at 0.0005, and the models were trained for a total of 10 epochs. For each type of image, separate models were trained for landmark detection on short- and long-axis images, respectively.

### Performance Evaluation and Statistical Analysis

The trained model was applied to all test samples. All results were first visually reviewed to determine whether landmarks were missed or unnecessarily detected (further details are described in Appendix E5 [supplement]).

The detection rate or success rate was computed as the percentage of samples with landmarks that were correctly detected. This rate was the ratio between the number of images with all landmarks detected and the total number of tested images. For all samples with successful detection, the Euclidean distance between the detected landmarks and assigned labels was computed and reported separately for different section views and different landmark points. Results from model detection and manual labeling were compared, and the Euclidean distance between the findings of the two readers was reported.

The detected key points were further processed to compute two derived measurements: the angle of the A-RVI to the C-LV for short-axis views and the LV length for long-axis views, the latter of which was computed as length from the detected apical point to the midpoint of two valve points (32). The model-derived results were compared with the manual labels. The results of the first reader were compared with those of the second reader to obtain references for interreader variation.

Results are presented as means ± standard deviations (instead of standard errors). A paired *t* test was performed, and *P* < .05 was considered to indicate a statistically significant difference (Matlab R2017b, MathWorks).

To test the sensitivity of detection performance in terms of the size of the Gaussian kernel used to generate the heatmap, two additional models were trained for long-axis cine images, with σ values equaling 6.0 and 2.0 pixels. Detection performance was compared across different kernel sizes for cine long-axis test images.

To visualize the characteristics of what trained models learned from each image, a saliency map was computed as the derivative of the CNN loss function with respect to the input image. A higher magnitude in the saliency map indicates that the corresponding image content has more impact on the model loss and indicates that the CNN model learned to weight those regions more heavily.

The cine long-axis test datasets were further split according to the scanner field strength. The Euclidean distances were compared for 3-T and 1.5-T scanners.

### Model Deployment

To demonstrate the clinical relevance of landmark detection of CMR, an inline application was developed to automatically measure the LV length from long-axis cine images on the MRI scanner. The trained long-axis model was integrated with MRI scanners by using the Gadgetron InlineAI toolbox (15). Although the imaging was ongoing, the trained model was loaded, and after the cine images were reconstructed, the model was applied to the acquired images as part of the image reconstruction workflow (inline processing) at the time of imaging. The resulting landmark detection and LV length measurements were displayed and available for immediate evaluation prior to the next image series being obtained. Figure E4 (supplement) provides more information for this landmark detection application. This example can be viewed in the Movie. Appendix E6 (supplement) provides additional information on model deployment and processing times.

**Movie:** Screen snapshot of inline landmark detection from a MR scanner. Original cine image series and detected landmarks are shown in the upper row. The estimated global longitudinal shortening ratio is estimated for every cardiac phase and plotted as a curve for reporting on the scanner.

The source files used to train the model are shared at *https://github.com/xueh2/CMR_LandMark_Detection.git*.

## Results

### Model Landmark Detection Rates

**Figure 4:**
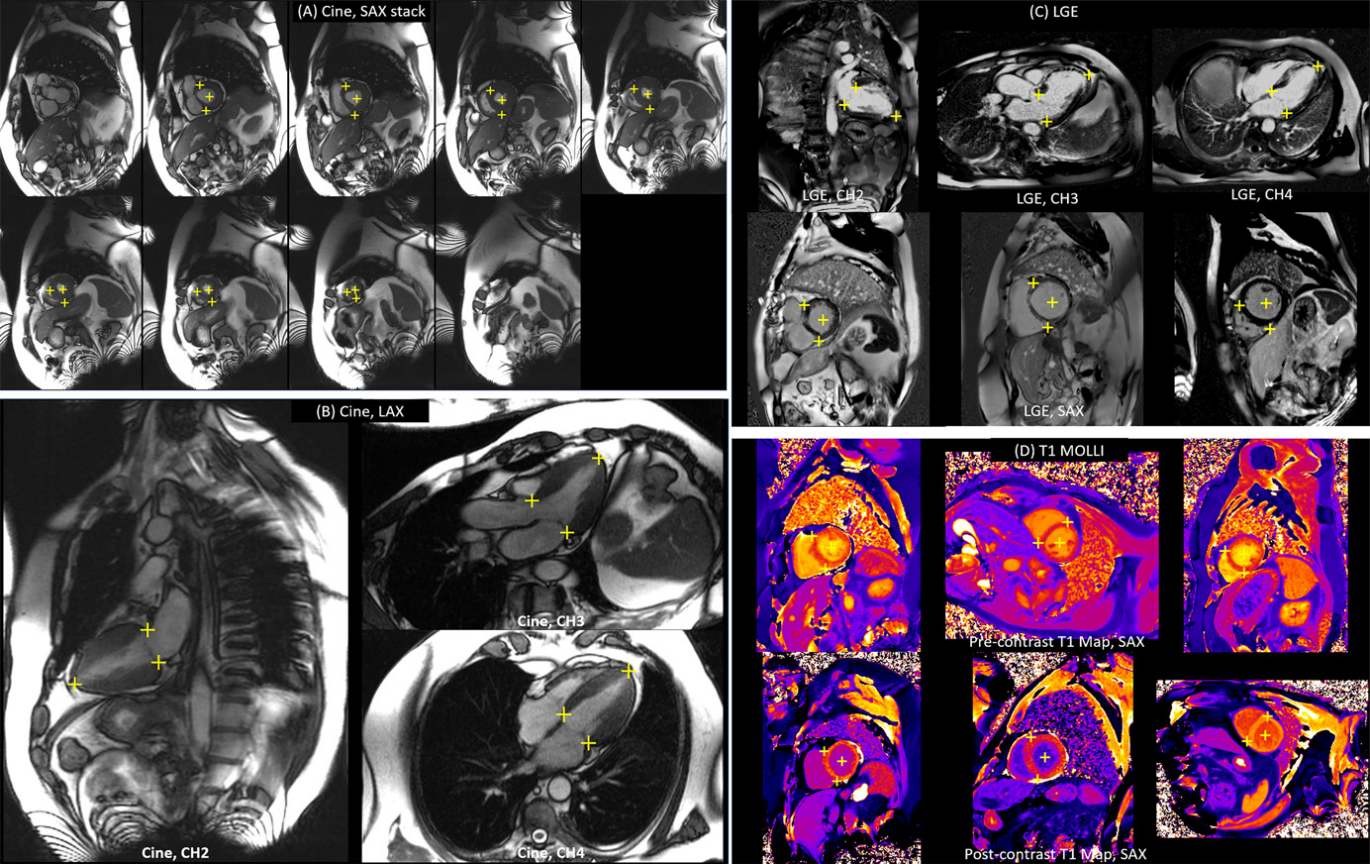
Examples of landmark detection. The left panels show landmark detection on **(A)** long-axis (LAX) and **(B)** short-axis (SAX) cine images. The right panels are examples of detection on **(C)** late gadolinium enhancement (LGE) and **(D)** T1 mapping modified Look-Locker inversion recovery (MOLLI) images. CH2 = two chamber, CH3 = three chamber, CH4 = four chamber.

The trained model was applied to the test datasets. Examples of landmark detection for different long-axis and short-axis views (Fig 4) demonstrate that the trained model was able to detect the specified landmarks. Table 2 summarizes the detection rate for all views and sequences on the test dataset. For the cine, the model successfully detected landmarks on 99.8% (2072 of 2076 images; no false-positive findings) of the two-chamber, three-chamber, and four-chamber long-axis images and on 96.6% (2906 of 3008 test images; 24 false-positive findings) of the short-axis images. For the LGE, the model successfully detected landmarks on 99.4% (1105 of 1112 images; two false-positive findings) of all long-axis views and on 97.6% (1056 of 1082; 11 false-positive findings) of all short-axis views. For T1 mapping, the model successfully detected landmarks on 98.7% (439 of 445 images; no false-positive findings) of the images.

**Table 2:**
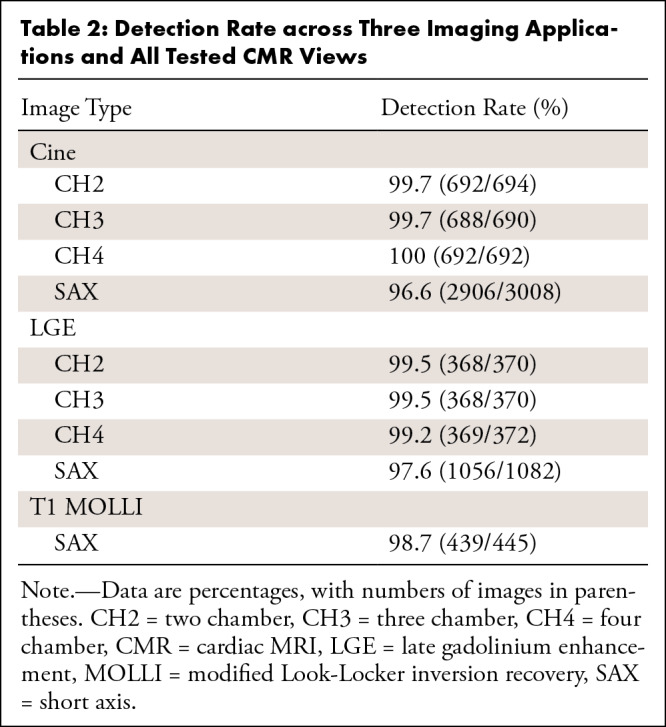
Detection Rate across Three Imaging Applications and All Tested CMR Views

The few failed detections on long-axis test images were due to incorrect imaging planning, unusual LV shapes, or poor image quality. Examples and a discussion of failed detections on long-axis images can be found in Figure E2 (supplement).

For the 102 mislabeled short-axis images acquired using cine imaging, the A-RVI was missed on 51, the P-RVI was missed on 25, and the C-LV was missed on 13. Half of the errors were found to be on the most basal and apical sections (defined as top two sections, or the last section for a short-axis series). For the 26 mislabeled short-axis images acquired by using LGE imaging, the A-RVI was missed on seven, the P-RVI was missed on one, and the C-LV was missed on two. A total of 11 errors were due to unnecessary landmarks being detected in sections outside the LV area. All detection failures on T1 MOLLI images (six of 445 test images) were failures to detect the P-RVI, which was due to unusual imaging planning for one patient. Examples of mislabeled short-axis cases can be found in Figure E3 (supplement).

### Euclidean Distances between Readers and the CNNs

**Figure 5:**
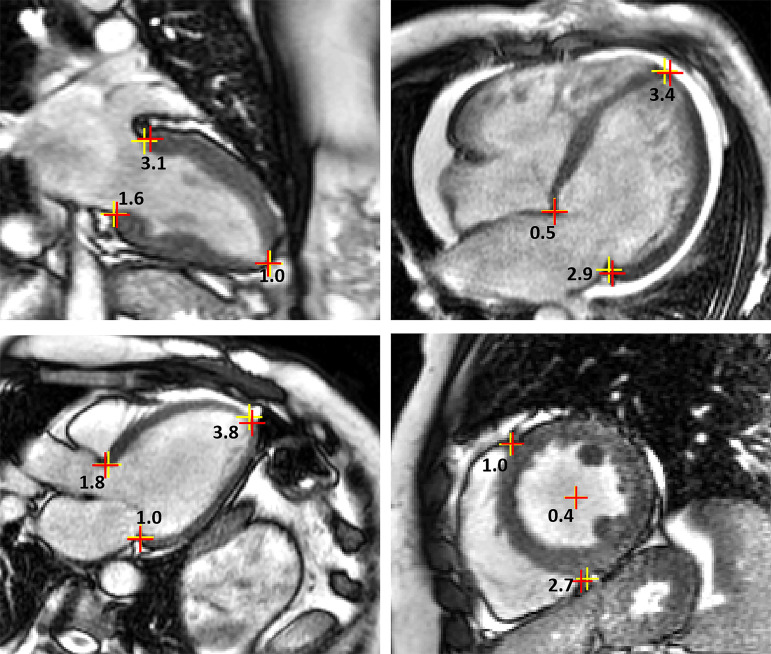
Examples of landmark Euclidean distances. For every pair of manually delineated (red crosses) and model-delineated (yellow crosses) landmarks, the distance (in millimeters) is labeled.

For all images on which detection was successful, the Euclidean distances between the model-assigned labels and the expert-assigned labels were computed. Tables 3 and 4 show the Euclidean distances and two derived measurements, which were reported separately for all imaging views and imaging sequences. The distances between the trained model and the first reader ranged from 2 to 3.5 mm. Figure 5 shows detection examples with model-derived and manually derived landmarks and their Euclidean distances, which demonstrate that the model-derived landmarks were in close proximity to the manually assigned labels. The mean Euclidean distances ± standard deviations for the long-axis cine and LGE images were 2.5 mm ± 1.9 and 3.0 mm ± 2.4. For the short-axis views, the mean Euclidean distance (across all landmarks) for cine, LGE, and MOLLI images were 2.5 mm ± 1.8, 2.4 mm ± 2.5, and 2.2 mm ± 2.0, respectively.

**Table 3:**
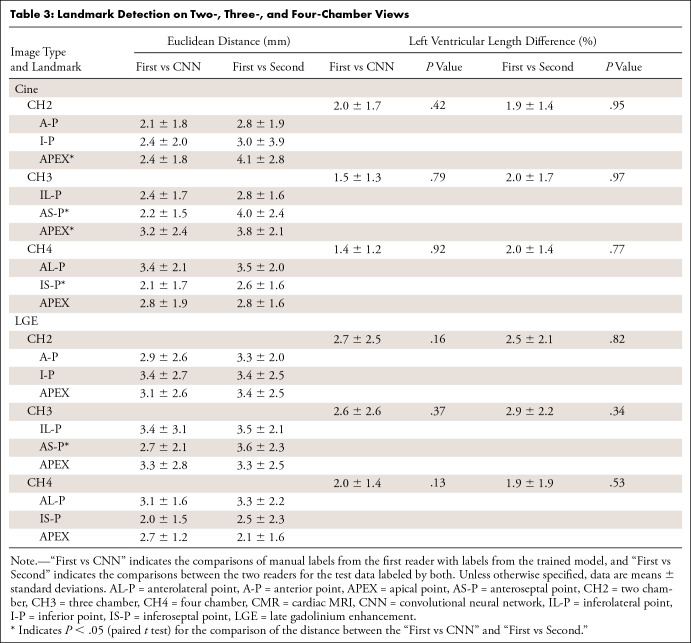
Landmark Detection on Two-, Three-, and Four-Chamber Views

**Table 4:**
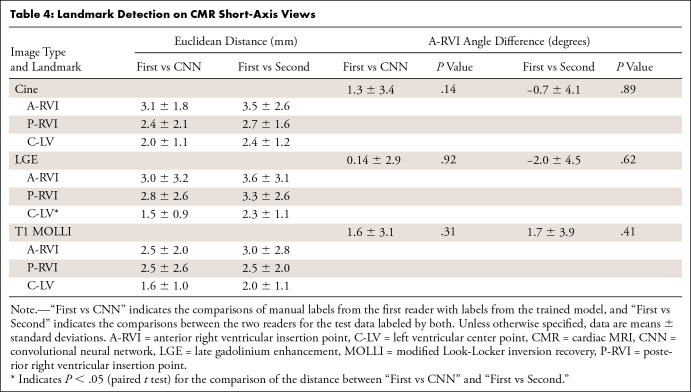
Landmark Detection on CMR Short-Axis Views

Tables 3 and 4 list the Euclidean distances between the findings of the two readers for the labeled portion of the test data. The Euclidean distances between the findings of the two human readers were comparable with the model distances. We found no evidence of differences between the A-RVI angle and LV length measurements provided by the trained models and those provided by the first reader for all imaging applications and imaging views. For the test data labeled by both readers, no differences were found between the findings for the two readers for either measure. The long-axis cine test images were split according to the acquired field strength (1.5 T, 1668 images; 3 T, 408 images). The mean distance ± standard deviation was 2.5 mm ± 1.6 for images acquired at 1.5 T and 2.3 mm ± 1.5 for images acquired at 3 T (*P* < .001).

The model was retrained with two more different Gaussian kernel sizes (2.0 and 6.0 pixels) for the long-axis cine datasets, bracketing the 4.0-pixel design to determine the sensitivity to the kernel size. The mean distances to the manually assigned landmarks from the first reader were 2.3 mm ± 1.6 and 2.2 mm ± 1.6 for models trained with σ of 2.0 and 6.0 pixels, and no differences were observed when compared with a σ of 4.0. The LV length was estimated for σ of 2.0 and 6.0 and showed no differences compared with measurements performed by the experts (*P* > .2 for all views). Figure E5 (supplement) provides an example of landmark detection with computed probability maps for three models, which shows that the detection was insensitive to Gaussian kernel sizes.

## Discussion

This study presents a CNN-based solution for landmark detection in CMR. Three CMR imaging applications (cine, LGE, and T1 mapping) were tested in this study. A multitask learning strategy was used to simplify training and ease deployment. Among images from the entire training dataset, the majority (86%) were cine images. As a result, a transfer learning strategy with fine-tuning was applied to improve the performance of the LGE and T1 mapping detection. The resulting models had high detection rates across different imaging views and imaging sequences. An inline application was built to demonstrate the clinical usage of landmark detection to automatically measure and output LV length on the MRI scanner.

Landmark detection by using deep learning has not been extensively studied for CMR but has been investigated for computer vision applications, such as facial key point detection (33,34) or human pose estimation (24,35). In these studies, two categories of approaches were explored for key point detection. First, the output layer of a CNN explicitly computed the x–y coordinates of landmark points, and L2 regression loss was used for training. Second, landmark coordinates were implicitly coded as heatmaps. In this context, the detection problem was reformulated as a segmentation problem. In human pose estimation, the segmentation-based models outperformed regression models (24,36). Here, fewer landmarks were detected and were more sparsely distributed spatially. The human pose images had much more variation than images of human faces, which often had been preprocessed as frontal position (37). It is easier for heatmap detection to handle landmark occlusion. For example, in Figure 1, some images may not include the targeted landmarks, which is represented by a low probability of detection outputs. For these reasons, this study adopted the segmentation model for CMR.

A recent study used heatmap landmark detection in the context of automated image plane prescription for CMR (38). This study trained a heatmap detection model on 892 long-axis and 493 short-axis steady-state free precession cine images. The midvalve plane and apical points were automatically detected and compared with manual localization, with a mean distance of approximately 5–7 mm. A recurrent U-Net architecture was used in another study to perform myocardial segmentation and detection of mitral valve and RV insertion points from cardiac cine images in one forward pass (39). This neural network was trained on 6961 long-axis images and 670 short-axis images. The detection distance was 2.87 mm for the mitral valve points and 3.64 mm for the RV insertion points.

Another study developed a patched fully convolutional neural network to detect six landmarks from cardiac CT volume (40). The training was performed on 198 CT scans, and the resulting average Euclidean distances to the manual label were 1.82–3.78 mm. Compared with previous studies of cardiac landmark detection, the current study curated larger datasets and detected more landmarks in cine, as well as LGE and T1 maps that had substantially different contrasts, to enable automated reporting and measurement of global longitudinal shortening. Detection was slightly less accurate on basal and apical short-axis sections. In these regions, the “ambiguity” of anatomy increases, leading the model to demonstrate more variance in data labeling and more difficulties with providing the correct inference. Additional discussion can be found in Appendix E7 (supplement).

There were limitations to this study. First, a single reader labeled the entirety of the datasets. Because of limited research resources, the second reader only labeled a portion of the test set to measure interreader variation. Second, three imaging applications were tested in this study. If the model were to be applied to the detection of a new anatomic landmark (eg, the RV center), imaging sequence, or cardiac view, more training data would be required. The use of transfer learning would reduce the amount of new data needed. The development process would have to be iterative to cover more imaging sequences and anatomic landmarks. Third, the data used in this study were collected from a single MRI vendor (Siemens). A recent study (41) reported that the performance of deep learning models trained on imagers from one vendor may decrease when used on imagers from different vendors, although augmentation was used to improve robustness. Further validation will be required to extend the proposed CNN models for use with CMR imagers from other vendors. It is very likely to require further data curation and training. Fourth, because of the availability of different imaging sequences, not all imaging sequences were performed across both of the included institutions, which limits the evaluation of generalizability across hospitals. We expect that the on-scanner deployment could enable the proposed models to be used in more hospitals and that further studies could provide more comprehensive datasets. Other limitations are related to preprocessing. Although the selected processing field of view of 400 mm^2^ has been large enough to cover the heart in our imaging experience, it is possible that an even larger configuration may be needed if the imaging planning is far off center. The model can be retrained with an even larger field of view, but the inline detection-result feedback could be used to alert readers to the need for adjustment or repeat acquisition.

In this study, a CNN-based solution for landmark detection on CMR was developed and validated. A large training dataset of 2329 patients was curated and used for model development. Testing was performed on 531 consecutive patients from two centers. The resulting models had high landmark detection rates across different imaging views and imaging sequences. Quantitative validation showed that the CNN’s detection performance was comparable with the interreader variation. On the basis of the detected landmarks, the RV insertion points and LV length can be reliably measured.
